# The Contribution of Executive Functions When Reading Multiple Texts: A Systematic Literature Review

**DOI:** 10.3389/fpsyg.2021.716463

**Published:** 2021-09-27

**Authors:** Christian Tarchi, Costanza Ruffini, Chiara Pecini

**Affiliations:** Department of Education, Languages, Intercultures, Literatures and Psychology, University of Florence, Florence, Italy

**Keywords:** executive functions, working memory, inhibition, shifting, multiple-documents comprehension, multiple-texts comprehension

## Abstract

In the present-day knowledge society, people need to critically comprehend information across multiple sources that express diverse and contradictory viewpoints. Due to the complexity associated with this process, an important role can be played by Executive Functions, that is, cognitive control processes used to regulate mental functioning and behavior when automatized elaborations are not sufficient. The aim of this article is to review existing research on the roles of executive functions when reading from multiple texts. To identify the appropriate studies, we conducted a search in the following databases: Web of science, Scopus, PsycInfo, Eric. The search string was created by combining the terms used in past literature reviews on executive functions and multiple-texts comprehension. From the total number of 4,877 records identified, seven articles met all the inclusion criteria and were analyzed. Given the scarcity of studies on the topic, we decided to examine also eight articles reporting indirect evidence about the association between executive functions and multiple-text comprehension. Our review revealed that the study of the association between executive functions and multiple-texts comprehension is underdeveloped. The results seem to suggest that working memory is involved in surface comprehension, whereas results about sourcing and intertextual integration processes are mixed. Indirect evidence suggests that other executive functions, such as planning or monitoring, may be involved when learning from multiple texts. More research on this topic is needed given the increasing complexity of the contexts in which reading activities take place.

## Introduction

In the present-day knowledge society, people have access to a great wealth of knowledge to solve information-based problems on relevant aspects of life (Bråten et al., 2011). From a social perspective, to make decisions people need to critically comprehend information across multiple sources that express diverse and contradictory viewpoints. At the same time, from an educational perspective, comprehending, and critically analyzing school texts are essential for academic success and lifelong learning (Li et al., 2016).

Due to the complexity associated with synchronously processing, evaluating, and integrating information from multiple texts for a specific purpose, several psychological processes are involved. Among these, an important role can be played by Executive Functions as they are cognitive control processes used to regulate mental functioning and behavior when automatized elaborations are not sufficient (Diamond, 2013).

While there are a plethora of studies exploring the association between Executive Functions and reading comprehension, much of the existing work focuses solely on the comprehension of single texts, with only a few studies have systematically examined this association when readers are engaged with multiple texts (Follmer and Sperling 2020). The original aim of the article was to review existing research on the roles of executive functions when reading from multiple texts. However, the scarcity of research on the topic made the literature review only about working memory with only a few studies available. Thus, the present article will present direct and indirect evidence discussing the contribution of working memory on multiple-text comprehension with the purpose of suggesting future lines of research that are needed.

### Multiple-Text Comprehension

Multiple-text comprehension involves the “building of a coherent mental representation of an issue from the contents of multiple documents that deal with the same issue from different perspectives” (Bråten et al., 2013, p. 322–323). Certainly, multiple-text comprehension is based on single-text comprehension. Readers need to comprehend each document, to identify information uniquely presented in single texts as well as overlapping information presented in different texts (Bråten et al., 2011). Multiple-text comprehension requires two additional mental representations: the integrated situation model and the intertext model (see the Documents Model, Britt et al., 1999; Perfetti et al., 1999). The integrated situation model defines a global representation of the situation described in the texts, resulting from the integration of the situation models for each single text. This is achieved through intertextual integration, defined as “connecting, combining, or organizing information from different texts to achieve diverse aims such as meaning-making, problem-solving, or creating new texts” (Barzilai et al., 2018, p. 976). Intertextual integration is a core element when reading and writing from contradictory texts, as it means going beyond the perspectives presented in the texts and elaborating a coherent approach (De La Paz and Felton, 2010; Mateos et al., 2011, 2018; Kobayashi, 2015). The intertext model represents relevant information about the source and how it connects to the text content and across texts. This is achieved through sourcing, defined as “identifying and representing source features to predict, interpret, and evaluate documents' content and relevance according to a reading task” (Brante and Strømsø, 2018, p. 777). It describes the processes into play when readers attend to, evaluate and use source information to understand the content (Bråten et al., 2018). Several studies suggest that sourcing skills are associated with multiple-text comprehension (Bråten et al., 2009; Wiley et al., 2009; Gil et al., 2010; Goldman et al., 2012; Anmarkrud et al., 2014; Barzilai and Eshet-Alkalai, 2015; Barzilai et al., 2015).

Over the last decade, multiple-text comprehension has received much attention from scholars, who produced several theoretical models. Recently a special issue on “Models of Multiple-Text Comprehension” was published in the journal Educational Psychologist (Volume 52, Issue 3, 2017). It includes four theoretical models for understanding the interrelations among multiple-text comprehension components (List and Alexander, 2017b). Richter and Maier (2017) examined the role of prior knowledge and beliefs in students' processing of multiple texts that agree or disagree with their initial positions. According to this theory, prior beliefs are used to validate sources, leading to one-sided mental models of controversial issues. However, if particularly motivated, readers can engage in strategies to prevent biasing effects of prior beliefs and deeply process the texts.

Braasch and Bråten (2017) explained how the introduction of conflict across texts induces readers to strategically shift their attentional resources toward constructing a mental representation of the messages that also includes source features.

List and Alexander (2017a) consider how learners approach multiple-text comprehension with a default stance toward task completion. Such default stance is determined by the interaction between their level of affective engagement with the topic discussed in the texts and their habits with regard to text evaluation (behavioral disposition).

Rouet et al. (2017) frames multiple-text comprehension as a problem-solving activity. In particular, the model describes the influence of the context and the task on multiple-text comprehension. Readers' approach to the multiple-text comprehension depends on their representation of contextual demands and opportunities (i.e., context model), which in turn influence initial goals and actions (i.e., a task model). Goals and actions influence reading processes and outcomes.

In an attempt to integrate different theoretical frameworks, List and Alexander (2019) presented the integrated framework of multiple text use, which conceptualizes multiple text use as unfolding through a series of three stages: establishing a stance toward task completion (preparation), activating behavioral, cognitive, and metacognitive/regulatory strategies while processing the texts (execution) and transforming the cognitive and affective outcomes gained by reading multiple texts into external products (production).

### Executive Functions

Executive Functions (EF) are a complex construct including several abilities and described by different theoretical frameworks (Miyake and Friedman, 2012).

Although multi-componential models define the main EF components differently (e.g., Miyake et al., 2000; Diamond, 2013; Friedman and Miyake, 2017; Morra et al., 2018), they agree on the existence of three main EF processes that are the base of more complex EF. *Updating* (Miyake et al., 2000) and *working memory* (Diamond, 2013) refers to the ability to manipulate verbal or visual information temporarily held in mind. *Inhibition* is the ability to inhibit automatic responses, compelling thoughts and behavior, and resist distractor interference, allowing one to focus on relevant information to reach a goal. Finally, *shifting* (Miyake et al., 2000) *and cognitive flexibility* (Diamond, 2013), refers to the ability to rapidly change tasks, operations, mental sets, or strategies to adapt to challenging and variable requests. These basic EFs, independent but connected, are fundamental for more complex EF such as problem-solving, abstract reasoning and planning, processes that are considered crucial for fluid intelligence (Diamond, 2013) and the core of sequential models of EF.

Sequential models describe executive functioning as a macro construct within a problem-solving framework that delineates separate and sequential phases that lead from the recognition of a problem to its solution (Bransford and Stein, 1993). Although approaches differed for the emphasis given to each phase, the problem-solving framework of EF identifies four main steps: problem representation, action planning, execution, and evaluation (Zelazo et al., 1997). To solve a problem, one must first construct a representation of the problem and the possible solutions, plan strategies by means-end analysis and time sequencing, keep the plan in mind and guide behavior during its execution and finally evaluate whether the goal (solution) has been obtained. Instead of measuring different EF components in isolation, the sequential model predicts that different EF processes (e.g., updating information in memory) can be engaged at various degrees in each problem-solving phase. One of the paradigms to study problem-solving is the think-aloud method. The method is useful to explore, instead of the outcome, the personal way of reasoning in constructing solutions and justifications to a problem (van Someren et al., 1994). Specifically, since problem-solving is described as a sequential process (Zelazo et al., 1997), the think-aloud method can explain almost every step taken by the problem solver.

Both types of EF model, fractionated and sequential, have been extensively used in developmental and educational psychology, bringing strong evidence on the connection between EF and several learning skills across different ages (D'Amico and Passolunghi, 2009; Swanson et al., 2009; Borella et al., 2010; Mammarella et al., 2010; Christopher et al., 2012; Bull and Lee, 2014; Viterbori et al., 2015; Zelazo and Carlson, 2020). Given the long-lasting plasticity of the neural circuits underpinning EF, the strength of the link with learning skills is likely bidirectional. Not only good EFs promote learning, but being engaged in complex learning tasks may be a life-long challenge of these important processes of cognitive control.

### Executive Functions and Reading Comprehension

Research on single-text comprehension has produced interesting results about the role played by EFs, in particular on working memory, inhibition and shifting (Butterfuss and Kendeou, 2018). Working memory was investigated by several studies that confirmed its importance in reading comprehension (Butterfuss and Kendeou, 2018). Specifically, readers must keep relevant information and exclude irrelevant information in working memory to successfully construct a coherent representation of a text (Palladino et al., 2001; Carretti et al., 2005). However, a few studies have not found an association between working memory and reading comprehension (Muijselaar and de Jong, 2015), a result that may depend on the fact that updating while reading is a far more demanding task than updating as assessed by standardized tests. Fewer studies investigated visuospatial working memory notwithstanding the literature points to the relevance of cross-modal working memory in reading task (Garcia et al., 2019) and to the role of visuospatial deficits in text comprehension impairment (Mammarella et al., 2015).

Inhibition was confirmed to be involved in the deactivation of irrelevant information (Butterfuss and Kendeou, 2018), which in turn helps the working memory not to be overloaded by the effects of distracting, outdated, or irrelevant information (Hasher et al., 1999; Christopher et al., 2012). Results however are mixed, which may depend on the specific type of inhibition assessed: resistance to proactive interference, prepotent response or active inhibition, and resistance to distractor inhibition (Borella et al., 2010). Less-skilled readers seem to have difficulty controlling irrelevant information at retrieval, but not at encoding (Borella et al., 2010).

Concerning shifting, the evidence supporting a relationship with reading comprehension is limited (Butterfuss and Kendeou, 2018). The few studies conducted suggest an involvement, especially in less-skilled readers (Cartwright, 2015; Guajardo and Cartwright, 2016). For instance, shifting may be involved when readers need to switch between reading strategies, monitoring one's comprehension, and engaging in metacognitive processes.

Overall only a recent study took into account different components of EF simultaneously and found that while working memory and inhibition play a relevant role in reading speed during decoding, cognitive flexibility and fluid intelligence are mainly related to reading comprehension (Johann et al., 2020).

According to Follmer's meta-analysis (Follmer, 2018), overall the studies on EFs and reading comprehension supported a moderate positive association. Besides working memory, inhibition, shifting, planning, sustained attention, and monitoring too were associated with reading comprehension.

Of notice, existing studies have been conducted on samples of different age groups, reporting mixed results within overlapping age groups (Butterfuss and Kendeou, 2018). Overall, the association between EFs and reading comprehension seems stronger in 6–17 years old readers and slightly weaker among adult learners (Follmer, 2018).

### Executive Functions and Multiple-Text Comprehension

Most of the theoretical model of multiple-text comprehension have not explicitly addressed the involvement of working memory (or other executive functions). An exception is represented by Richter and Maier (2017). According to the authors, working memory is a critical resource in promoting strategic and elaborative processing of conflicting information in multiple texts.

In a recent theoretical chapter, Follmer and Sperling (2020) reviewed the literature on multiple-text comprehension to derive indications about the hypothetical involvement of EFs. Working memory is supposed to be involved in the selection of task-relevant sources, integration of information across texts and between text content and prior knowledge. Thus, working memory is supposed to assist the reader in constructing an integrated situation model by facilitating inference generation as well as information evaluation and integration.

Follmer and Sperling also conceptualized the involvement of inhibition and shifting. As inhibition is involved in the suppression of task-irrelevant textual information, it is theorized to have a primary role in relevance processes, that is, the extent to which information is consistent with the task (Rouet and Britt, 2011). As shifting is involved in the ability to move flexibly across sources, it is theorized to be involved in readers' ability to create connections across texts, make sense of conflicting information, and construct valid intra- and inter-textual inferences.

These hypotheses can be mapped onto multiple-text comprehension. Braasch and Bråten discrepancy-induced source comprehension model 2017 suggests that discrepancy may activate shifting, which in turn helps the reader to direct attentional resources toward source features. Working memory should contribute to determine the reader's behavioral disposition in the multiple-text comprehension task (List and Alexander, 2017a). Working memory should facilitate the implementation of the skills necessary to evaluate sources and to integrate texts. In turn, a high level of behavioral disposition can induce an appropriate default stance to the multiple-text comprehension task (evaluative or critical analytic). Rouet et al. RESOLV model 2017 postulates the contribution of planning and self-regulation skills as EFs involved in the construction of the task model and in the activation of appropriate reading processes. These EFs may support the reader to move along the task model, making different decisions or engaging in different actions, and redefining the task.

### Rationale and Objective of the Review

Prior studies have hypothesized that EFs play an important role in comprehension processes as they support the understanding of both texts and perspectives (e.g., Kendeou et al., 2013; Georgiou and Das, 2016; Follmer, 2018). Overall, the present literature review aims at identifying associations between executive functions and the processes involved when reading multiple texts.

The research questions of the present study are:

1) What is the state of art of research on the association between executive functions and multiple-text comprehensions? Which executive function has received more attention, and which has been neglected?
2) What is the contribution of working memory to the different processes involved in multiple-texts comprehension processes?
3) Are there trends in data as a function of age (developmental trends)?

Results from the literature review will contribute to design a theoretical framework of multiple-text comprehension based on the involvement of EFs, as well as identify the open issues and needs for future research.

## Method

### Search Methodology

To identify the appropriate studies, we conducted a search in the following databases: Web of science, Scopus, PsycInfo, Eric. We inserted relevant terms in the keywords field. The complete search string was:

KW (executive function* OR executive control OR cognitive control OR behavioral control OR self-control OR effortful control OR self-regulat* OR regulat* OR inhibition OR working memory OR updating OR cognitive flexibility OR shifting OR goal planning OR monitoring OR sustained attention OR prefrontal cortex)

AND

KW (multiple source* OR multiple text* OR multiple document* OR primary document* OR primary source* OR information source* OR information problem solving OR information skill* OR digital competenc* OR digital skill* OR KW digital literacy OR e-skill* OR source evaluation OR critical literacy OR search strategies OR sourcing).

This search string was created by combining the terms used in past literature reviews on EFs (Burin et al., 2015; Craig et al., 2016; Friedman and Miyake, 2017; Follmer, 2018; Pereira et al., 2018; Kassai et al., 2019) and multiple-texts comprehension (van Laar et al., 2017; Barzilai et al., 2018; Brante and Strømsø, 2018; Primor and Katzir, 2018). The literature search was conducted in April 2020. The literature search was repeated in February 2021 to control for articles published since when the search was first performed. We included filters for document type (peer-reviewed articles only) and language (English language only). This method provided 3,478 results.

We integrated this search method with a snowball technique, that is we added reference lists of the literature reviews used to identify search terms and previously listed. This method provided 1,398 results.

We integrated the results from the searches conducted in the four databases with the reference lists obtained through snowball search to identify duplicates. From the total number of 4,877, 583 duplicates were identified and removed, reducing the number of references to examine for inclusion to 4,294.

### Inclusion Criteria

Studies had to meet all the criteria outlined below to be eligible for inclusion in the analysis:

1. Articles published in English in peer-reviewed journals;
2. Articles reporting empirical studies (with any research design or methodology);
3. Articles discussing the topic of EFs and comprehension of two or more texts;
4. Participants were (or included) samples from only non-clinical populations (e.g autism, ADHD, learning grade disorders, cognitive disabilities);
5. At least one measure of EF was included in the research design;
6. Participants were given a learning task with two or more texts involved;
7. Overall comprehension, sourcing or integration was measured.

First, we examined the results by titles and abstracts, mainly applying criteria 1–3. A total of 3,985 results were excluded. Then, we examined the full-texts of the remaining 309 results, mainly applying criteria 4–7, which led to the exclusion of 294 results: 276 results did not include a measure of EFs or multiple-text comprehension; one result did not investigate the association between EFs and multiple-text comprehension; 16 results did not report empirical studies, and one result was not published in English.

Out of the remaining 15 articles, seven articles met all the inclusion criteria and were analyzed as direct evidence for the association between EFs and multiple-text comprehension. Given the scarcity of studies on the topic, we decided to examine also eight articles reporting indirect evidence about the association between EFs and multiple-text comprehension.

**Figure 1:**
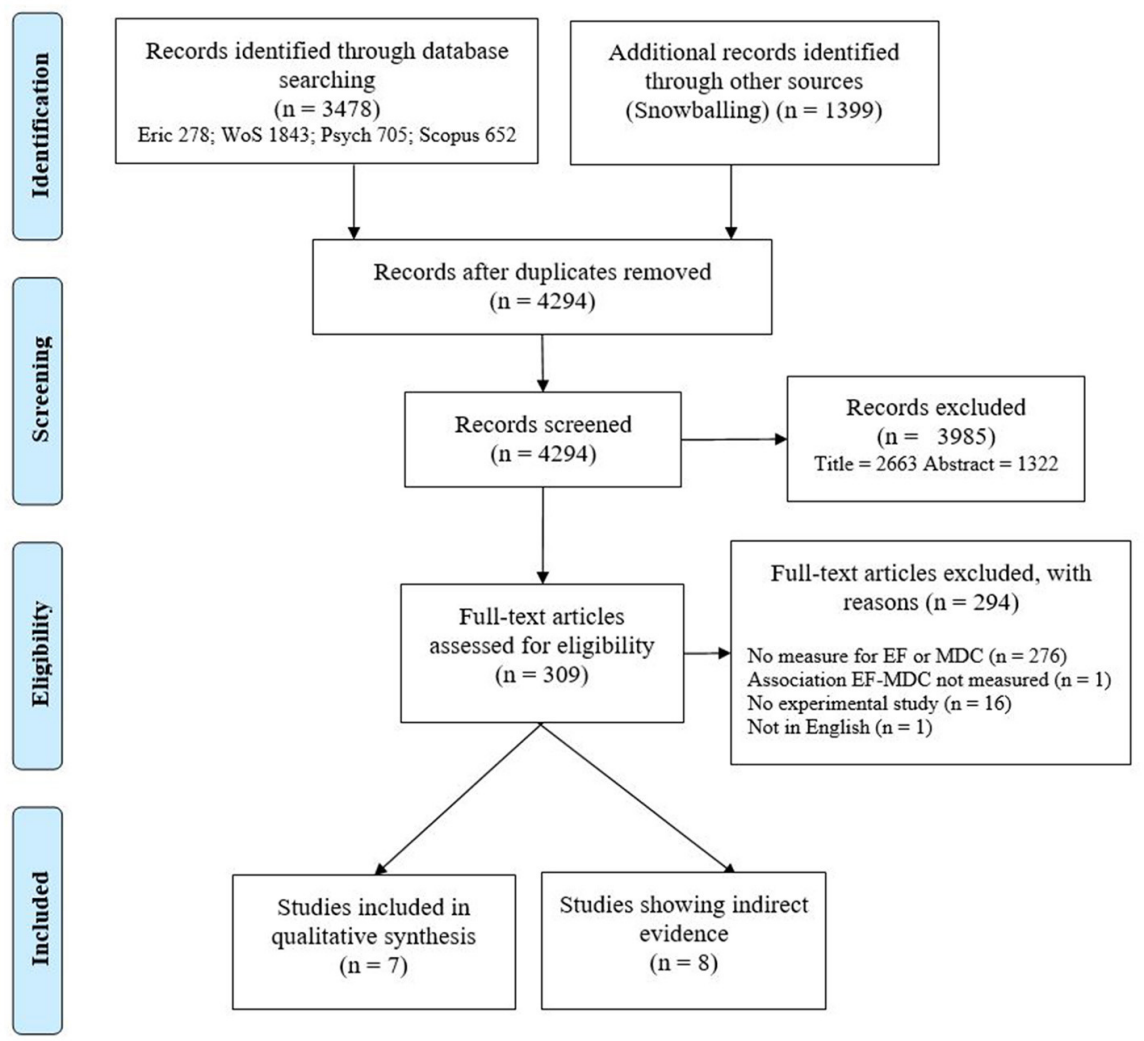
PRISMA flow diagram.

In each step (analysis of titles, abstracts, and full-texts), the results were coded by two authors of the present article and cases of disagreement were discussed until resolution. See Figure 1 for an overview of the process with the PRISMA method (Moher et al., 2009).

## Results

### Direct Evidence on the Association Between Executive Functions and Multiple-Text Comprehension

Seven studies presented evidence on the association between EFs and multiple-text comprehension (see Table 1).

**Table 1:** Studies presenting direct evidence on the association between executive functions and multiple-text comprehension.

**Study**	**Participants** **(*n* and school-level)**	**Executive functions**	**Multiple-text comprehension**	**Association**
Andresen et al. (2019)	*n* = 44 (high school, grade 10)	Working memory (Span task)	Reading three web pages + 2 integrative questions (intertextual integration)	Yes
Beker et al. (2019)	*n* = 105 (54 in grade 4; 51 in grade 6)	Working memory (Sentence span task)	Reading 20 expository pairs of texts + 3 questions (literal, open, applied) + process data (switchesand time)	Partial
Braasch et al. (2014)	*n* = 59 (high school, college-preparatory course)	Working memory (Span task)	Reading six paper documents + essay writing (scientific concepts included) + rank order task (evaluation and justification) + sentence verification task (intertextual inferences identified)	Yes
Florit et al. (2020)	*n* = 94 (primary school, 4th grade)	Verbal working memory (updating task)	Reading three different documents for each of two topics + essay writing (intertextual integration, valid inferences, source identification and use)	Partial
Latini et al. (2019)	*n* = 133 (undergraduates)	Problem solving (Cognitive reflection test)	Reading two texts + four integrative questions (intertextual integration)	Partial
Mason et al. (2018)	*n* = 72 (secondary school, grade 7)	Working memory (Reading span test)	Reading six texts + Rank ordering task (evaluation)	No
Mason et al. (2017)	*n* = 104 (secondary school, grade 7)	Working memory (Reading span test)	Reading six texts + sentence verification task (surface comprehension) + essay writing (references to sources and source-content links)	Partial

The selected studies were recently published (2014–2019) and analyzed performances in participants ranging from primary school to adulthood. All studies except for one analyzed working memory as an EF. The exception is represented by problem-solving. Concerning multiple-text comprehension measures, the studies explored comprehension, sourcing, and intertextual integration.

Andresen et al. (2019) compared 10th-grade students with and without dyslexia reading multiple digital texts. The participants were 44, 22 diagnosed with dyslexia and 22 without a diagnosis. The participants read three web pages about a controversial issue presenting two main perspectives. Following, participants were asked to respond (orally) to two integrative open-ended questions. Protocols were coded by intertextual integration and references to information from the three web pages: these two scores were multiplied to obtain a total multiple source integration score. Participants' working memory was assessed through a listening span task (Braasch et al., 2013; derived from Daneman and Carpenter, 1980): they listened to 12 sets of unrelated sentences, with sets gradually increasing from two to five sentences. For each trial, participants had to answer a comprehension question and remember the final words of each sentence. According to the results, overall students with better working memory performed better in the multiple source integration task [*F*_(1,38)_ = 4.50, *p* = 0.041, partial η^2^ = 0.106]. Word recognition had no statistically significant effect on the multiple source integration task, suggesting that differences in multiple-text comprehension should be attributable to working memory deficits, rather than decoding difficulties. As the focus of the present review is on non-clinical sample, of interest is that working memory seems to play a main role in intertextual integration in upper secondary school students, independently of basic literacy competences.

Braasch et al. (2014) investigated the effect of theories of intelligence on multiple-text comprehension in 59 students enrolled in college preparatory courses at a public upper secondary school. Participants had to read six documents on a socio-scientific topic (three more useful documents that were consistent with current scientific thinking and evidence and three less useful documents that included erroneous concepts). After reading, the participants were asked to (i) write an explanation essay (coded by the number of concepts from higher-quality documents that were included); (ii) rank-order the texts from most to least useful; (iii) explain why they assigned a specific rank to the texts (coded by reference to usefulness); answer to an intertextual inference verification task. The participants' working memory capacity was assessed through a similar listening span test to the one used in the previous study. According to the results, working memory was associated with students' abilities to discriminate between more and less useful documents (β = 0.41, *p* = 0.003) as well as their abilities to draw inferences across documents (β =.35, p =.015), but not with the inclusion of scientific concepts in the essays (β = 0.19, *p* > 0.05) nor ranking justifications (β = 0.06, *p* > 0.05). This study provides evidence for a partial involvement of working memory with documents selection and depth of elaboration across texts in upper-secondary school students.

Beker et al. (2019) investigated the spontaneous integration processes when reading multiple texts in young readers (105 children attending Grade 4 and 6). The participants read 20 expository text pairs, manipulated in a way that the second text included an internal inconsistency, which could be resolved by the first text in one condition (inconsistent-with-explanation) or not in the other condition (inconsistent-without-explanation). The condition was within-subject. After reading the texts, children were asked to answer a comprehension question (multiple-choice), a recall question (open), and an application question (open). The recall responses were coded by the origin of the information (first text, second text, both texts or prior knowledge), and the number of switches between texts. The application responses were coded by correctness (i.e., whether children used at least parts of the explanation in the first texts). The participants' working memory was assessed through a listening span task (Daneman and Carpenter, 1980), with a similar procedure to the one used in the two previous studies. According to the results, working memory did not correlate with reading times or integration in the recall question, but it was associated with total recall [*r* = 0.75, *p* < 0.001]. Working memory did not affect the performance in integrating when recalling or resolving inconsistencies in primary school children.

Florit et al. (2020) investigated the influence of word reading fluency, verbal working memory, comprehension monitoring, and single-text comprehension on multiple-document comprehension. The participants were 94 primary school children attending the fourth grade. They read three documents on each of two controversial topics. The documents contained partially overlapping and partially conflicting information. After reading the texts, the participants were asked to write two short essays, one for each topic. The essays were coded to assess the following processes: intertextual integration, number of valid inferences, the number of texts the children referred to in their written essays, the number of explicit references to sources and the number of source-to-content links. Verbal working memory was assessed using a memory updating task: the participants were given a list of six objects and had to write down the three nouns referring to the smallest objects. According to the results, verbal working memory was correlated with multiple-document comprehension for one of the topics only [*r* = 0.22, *p* < 0.05], but not for the other one [*r* = 0.20, *p* > 0.05] or number of source-content links [*r* = 0.11, *p* > 0.05]. However, in the regression analysis, verbal working memory was not a significant predictor of multiple-texts comprehension in primary school children.

Latini et al. (2019) investigated whether reader engagement and comprehension differ when readers work with identical printed vs. digital texts. The participants were 133 undergraduate students. They read two expository texts presenting two different perspectives on the topic of social media use. Two variables were manipulated between subjects: the reading purpose (reading texts to prepare for an exam vs. reading for pleasure) and the reading medium (reading both texts in print, reading both texts on screen, or reading one text in print and the other one on screen). After reading the texts, participants were asked four integrative questions. Responses were scored according to whether participants referred to information discussed in the texts and integrated information across texts. The use of connective words was also counted. Participants' problem-solving skills were assessed through the Cognitive Reflection Test (Frederick, 2005): the task included three numerical tasks for each of which the participants had to inhibit an automatic response to produce the correct response. According to the results, the cognitive reflection test performance correlated with text integration scores [*r* = 0.292, *p* < 0.01], but when included as a covariate in the inferential analysis its effect was not significant in any of the dependent variables. Problem-solving skills was not associated with multiple-texts comprehension in undergraduate students.

Mason et al. (2018) investigated the role of students' implicit associations related to mobile phones and psychophysiological self-regulation when evaluating online information about an unsettled topic in 72 secondary school students, attending grade 7. The participants read six pages presenting different positions on the topic of the potential health risks associated with the use of mobile phones. The sources varied in reliability. Information about the author, credentials, and date of publication (if available) of each website was included. After having read the text, the participants were asked to rank the six websites in order of reliability and provide a written justification for their rank-ordering. The participants received 1 point for each website correctly ranked. Concerning the justification task, the following categories were identified: source characteristics, personal opinion, reference to other sources, and reference to the content. Working memory capacity was assessed through a reading span test (Pazzaglia et al., 2000), using a similar procedure to the studies previously described. According to the results, working memory was not correlated with source evaluation [*r* = 0.22, *p* > 0.05] and was not a significant predictor when included in the regression model [β = 0.23, *p* = 0.06]. Thus, working memory was not associated with sourcing when reading multiple texts in secondary school students.

Mason et al. (2017) studied the role of students' dispositional emotion reactivity when comprehending conflicting online information on a controversial topic in 104 secondary school students, attending grade 7. The participants were assigned six online sources on the controversial topic of health risks related to the use of mobile phones. The texts differed in reliability. After having read the texts, the participants were asked to write an essay. The participants' essays were coded by sourcing (i.e., explicit references to the six source texts and the number of source-content links) and level of intertextual integration (i.e., the extent to which both positions are mentioned and argued). The participants were also given a sentence verification task to assess surface comprehension. Working memory capacity was measured with the same task as in the previous study (reading span test, Pazzaglia et al., 2000). According to the results, working memory correlated with surface comprehension [*r* = 0.27, *p* < 0.01], but it was not associated with either sourcing or intertextual integration when reading multiple texts.

### Indirect Evidence on the Association Between Executive Functions and Multiple-Text Comprehension

In this paragraph, we will review 8 articles that, despite having been excluded from the final selection, may provide indirect evidence for the association between EFs and multiple-text comprehension (see Table 2). The main reason for exclusion is that several studies did not measure directly multiple-texts comprehension, but they assessed related processes, such as answers to single text questions (Isberner et al., 2013), use of hypertexts (Banas and Sanchez, 2012), web searching strategies (Sharit et al., 2015; Moehring et al., 2016) or epistemic beliefs concerning the justification for knowing (Bråten and Ferguson, 2014). In other cases, EFs were not measured by specific tests as problem-solving strategies were inferred by think-aloud methods (Bråten and Strømsø, 2003; Hilbert and Renkl, 2008; Brand-Gruwel et al., 2009).

**Table 2:** Studies presenting indirect evidence on the association between executive functions and multiple-text comprehension.

**Study**	**Participants** **(*n* and school-level)**	**Executive function**	**Multiple-text comprehension**	**Association**
Banas and Sanchez (2012)	*n* = 62 (undergraduates)	Working memory (Automated operation span task)	Reading a website organized in a hierarchical tree structure + search questions + tree structure task (correct placement or matched terms)	Partial
Brand-Gruwel et al. (2009)	Study 1: *n* = 10 (5 PhD students and 5 undergraduates) Study 2: *n* = 15 (undergraduates) Study 3: *n* = 23 (secondary school, grade 9)	Think-aloud (Regulation)	Answering questions with information found on the Web + Think-aloud (time, frequency and sequencing of sub-skills)	Yes
Bråten and Ferguson (2014)	*n* = 120 (high school, grade 10)	Working memory (Span task)	Epistemic belief questionnaire	Yes
Bråten and Strømsø (2003)	*n* = 7 (undergraduates)	Think-aloud (Strategic processing)	Reading one self-selected text to be integrated with other personal sourcers + think-aloud (frequency of strategies)	Yes
Hilbert and Renkl (2008)	*n* = 38 (undergraduates)	Intelligence; Think-aloud (regulation)	Reading 6 documents and concept-mapping + learning questions (correctness and knowledge increase) + think-aloud	Partial
Isberner et al. (2013)	*n* = 77 (undergraduates)	Working memory (Reading span)	Reading two texts + sentence verification task + recognition task + plausibility judgment.	Yes
Moehring et al. (2016)	Study 1: *n* = 120 adults Study 2: *n* = 171 (high school)	Fluid intelligence (Fluid reasoning scale)	Computerized digital literacy test	Yes
Sharit et al. (2015)	*n* = 60 (adults)	Working memory (Alphabetic span); reasoning (inference test); processing speed (digit symbol substitution); executive function (trail making test)	Using the Internet to answer questions (Search time; number of websites visited; switches between websites; search accuracy)	Partial

Banas and Sanchez (2012) investigated if differences in working memory influenced digital text comprehension in 64 undergraduates. Participants read a website on the taxonomy of plants organized in a hierarchical tree structure meanwhile answering 18 search questions, used to examine subsequent performance changes. Before and after reading the digital texts, a hierarchical tree construction task and a matching terms task on biology and plants' topic were used to measure digital text comprehension. In detail, the matching task was used to assess simple explicit relationships between subunits and the provided term, whereas the tree construction task detected the ability to recover from memory the entire hierarchy. Automated operation span task (Unsworth et al., 2005) was used to assess verbal working memory. According to the results, the search questions' performances were not correlated with working memory [*r* = 0.04, *p* > 0.05]. Working memorydid not predict gains in matching task scores [*R*^2^ = 0.09, *F*_(4,57)_ = 1.37, *p* > 0.05] but predicted a significant portion of the variance in gain on the tree construction task [*R*^2^ = 0.18, *F*_(4,57)_ = 3.10, *p* < 0.05]. Thus, working memory was associated with the understanding the underlying structure of the site. Structure is a key component in text comprehension and becomes a challenge in the context of multiple-texts comprehension. Indeed, readers may have to integrate information across texts with complex and differing structures (for instance when reading a persuasive text, an argumentative one, and an informative text).

Brand-Gruwel and colleagues (Brand-Gruwel et al., 2009) studied the “information problem solving using internet model” (IPS-I-model) across different educational levels (study 1: 5 PhD students and 5 Psychology freshmen; study 2: 15 student teachers; study 3: 23 secondary education students). This model combines five skills to access and use the information on the internet (Eisenberg and Berkowitz, 1990; Brand-Gruwel et al., 2005): define information problem, search information, scan information, process information, organize and present information. In this model, regulation activities, such as orientation, monitoring, steering, and evaluation, have an important role in the execution of the skill, as the problem solver has to plan the way to solve the question. The information problem-solving process was explored using a think-aloud method. Participants were asked to solve the information problem and write a concept article (study 1), an outline (study 2) or a short answer twice (study 3) with video recording while using the internet; think-aloud expressions were translated into protocols and analyzed. According to the results, all students used the 5 constituent skills in an interactive way and regulative processes. Secondary education students, in contrast to PhD students, freshman and teacher students, showed a lower frequency of regulation activities [*z* = −3.23, *p* < 0.001; *z* = −2.64, *p* < 0.01] and subskills, that is to say monitoring and steering [*z* = −3.60, *p* < 0.00; *z* = −2.64, *p* < 0.01; *z* = −5.15, *p* < 0.001] and orientation [*z* = −2.87, *p* < 0.01; *z* = −2.83, *p* < 0.01; *z* = −4.19, *p* < 0.001]. Freshmen regulated less than the student teachers [*z* = −2.66, *p* < 0.01]. No differences between groups were found in the evaluation of sources and information [$\chi_{\left(3,\text{N}=46\right)}^{2}$ = 7.58, *p* < 0.05]. Regulation as an executive function seems to be involved in the processing of multiple texts as shown by participants' think-aloud. The study also supports the hypothesis of a development trend as older (and more skilled) participants showed a higher regulation activity than younger (and less skilled) participants.

Bråten and Ferguson (2014) investigated if beliefs in personal justification, justification by multiple sources and justification by scientific authority predicted science achievement in 120 school-aged children, taking into account working memory capacity and preferences for rationality. Students completed an epistemic belief measure (Justification for Knowing Questionnaire), the rationality scale of the Rational- Experiential Inventory (REI) and a verbal working memory span task (Swanson and Trahan, 1992). According to the results, working memory positively correlated with science achievement [*r* = 0.46, *p* < 0.001] and justification by multiple sources [*r* = 0.18, *p* < 0.05] and it negatively correlated with personal justification [*r* = −0.24, *p* < 0.01]. Working memory is involved with epistemic cognition, which in turn has been found to be intrinsically involved with multiple-texts comprehension (see Bråten et al., 2011). We can hypothesize an indirect influence of working memory on multiple-texts comprehension via the mediation of epistemic cognition in school-aged children.

Bråten and Strømsø (2003) explored processing strategies during reading expository texts, their changes over time and their link to academic achievement in 7 undergraduates. Students were asked to read one self-selected text and integrate it with other personal sources while thinking aloud. The task was performed three times in subsequent sessions. Reading, audio and video registration were recorded. The think-aloud protocol was implemented to identify five categories of strategic processing, i.e. memorization, elaboration, organization, evaluation, monitoring, that are known to be relevant for reading comprehension (Pressley and Afflerbach, 1995) and self-regulated processing (Weinstein et al., 2000). The authors distinguished between two types of monitoring activities: comprehension confirmation and problem detection and solving. Results showed that in each session students used more frequently the strategy of memorization and elaboration and less frequently that one of evaluation. Problem-detection/problem-solving was used more in the first session, and less in the third session while comprehension confirmation's strategy increased from the first to the third session. Students used different strategies when having to review a test: they used more comprehension confirmation and elaboration's strategies and less problem detection/problem solving and memorization's strategies. Students with better academic results used less memorization and elaboration's strategies and more organization and comprehension confirmation's strategies. When reviewing the test, they used more comprehension confirmation (monitoring) while students with low academic results used more elaboration and less problem detection/problem solving (monitoring) strategies. While the study did not directly measure multiple-texts comprehension outcomes, it provides evidence of strategic processing occurring during the reading phase, suggesting an involvement of executive functions when reflecting over the texts in undergraduate students.

Hilbert and Renkl (2008) explored cognitive processes involved in effective concept mapping, requiring to select important concepts and to link them, in 38 undergraduates. Participants read 6 articles on a specific topic and then produced a concept map. Two learning outcomes were measured: (i) a comprehension question in the pre-test, after reading the six articles and after producing the concept map to assess knowledge, (ii) a seven multiple choice questions post-test to assess intertextual integration. Participants used a think aloud protocol during concept mapping. Authors segmented the recorded protocols into verbal units codified with ten categories: elaboration of concepts, relevance, relationships, negative monitoring, positive monitoring, planning and controlling, false statements, activity report, reading aloud, non-content problems. The planning and control of the mapping process significantly correlated with the scores at the integration test [*r* = 0.57, *p* < 0.001] but not with knowledge increase [*r* = −0.01, *p* > 0.05]. Regulation, as assessed though think-aloud, was involved in intertextual integration, but not with an increase of participants' knowledge after reading the texts in undergraduate students.

Isberner et al. (2013) investigated the effects of the presence of graphs in texts on the perceived plausibility and situation model strength, which is the mental representation of texts' contents (van Dijk and Kintsch, 1983). Seventy-seven undergraduate students read two pairs of scientific conflictual texts. After reading each text, participants completed a verification task, a recognition task and a plausibility judgment task on 24 items. Verbal working memory was explored with a computer-based reading span test (Oberauer et al., 2000). According to the results, a strong positive main effect of working memory capacity on the situation model strength measured by the verification task was found [*F*_(1,69)_ = 14.2, *p* < 0.001, η^2^ = 0.17]. The study provides indirect evidence supporting an involvement of working memory in the construction of an integrated situation model in undergraduate students.

Moehring et al. (2016) explored the relationship between fluid intelligence, crystallized intelligence (general knowledge in study 1 and specific prior knowledge in study 2) and digital literacy in 120 adults (study 1) and 171 high school students (study 2). Fluid intelligence was measured with the figural reasoning scale and crystallized intelligence with the declarative knowledge items of the Berlin Test of Fluid and Crystallized Intelligence (Wilhelm et al., 2014). Digital literacy was assessed with a computerized digital literacy test which required using websites to answer 13 comprehension questions (multiple choice and short answer). In both studies, figural reasoning and declarative knowledge explained about 85% of the individual differences in digital literacy [$\chi_{\left(101\right)}^{2}$ = 107.2, *p* = 0.32]. Fluid intelligence was involved in the comprehension of multiple texts in adults and high school students, although it is not clear whether it contributes to the comprehension of single texts or on the construction of an integrated situation model.

Sharit et al. (2015) studied the capacity in using the web to resolve an information problem-solving task in 60 adults. Age, web experience, and several cognitive abilities (i.e., working memory, reasoning, processing speed, verbal ability, EF, visuospatial ability) were measured. Participants received a booklet with two health scenarios, and they were asked to use the Internet to answer a series of interpretative and decisional questions (indices of search accuracy). Search time, the number of distinct websites visited and the number of transitions between websites were recorded. Authors assessed verbal working memory with the alphabet span (Craik, 1986), reasoning with the inference test (Ekstrom et al., 1976), processing speed with the digit symbol substitution (Wechsler, 1981) and planning and cognitive flexibility with the trail making test (Reitan, 1958). Because of missing data, the results were analyzed only in the first scenario. Search accuracy significantly correlated with reasoning [*r* = 0.43, *p* = 0.001], processing speed [*r* = 0.28, *p* = 0.030] and planning [(Trails B) *r* = −0.28, *p* = 0.029] but not with working memory [*r* = 0.21, *p* = 0.10]. High processing-speed participants obtained higher search accuracy scores [*p* < 0.005] in comparison to low processing-speed participants. Groups with high planning and reasoning skills achieved higher search accuracy scores than those with lower scores [*p* < 0.008; *p* < 0.018]. This study involved science achievement as the outcome rather than the comprehension of multiple texts. Nevertheless, it provides some evidence supporting an involvement of executive functions (but not working memory) when searching for information in adults.

## Discussion

Learning from multiple texts is a fundamental competency in our society, as it guarantees a critical selection, elaboration and evaluation of texts discussing complex issues that are relevant for our lives. At the same time, learning from multiple texts is also a fundamental academic skill, as school assignments are increasingly based on students' ability to comprehend and synthesize information across texts. As several processes and levels of representation are involved in multiple-texts comprehension, EFs are expected to play an important role. For this reason, we conducted a systematic review to investigate this hypothesis. Unfortunately, our review revealed that the study of the association between EFs and multiple-texts comprehension is underdeveloped. We were able to identify only seven studies that fulfilled our criteria, but:

i) The majority investigated verbal working memory capacity, with only one study exploring problem-solving, surprisingly neglecting other EFs that are considered crucial for reading comprehension (e.g., inhibiting or shifting); moreover, no study investigated visuospatial or cross-modal EF nor measured different EF components simultaneously;
ii) All the studies treated the EF measured as a covariate, and not as an independent variable, thus limiting the potential of the data to significantly contribute to the involvement of EFs when reading multiple texts;
iii) The studies varied in population (e.g., secondary school or undergraduate students), constructs (e.g., intertextual integration or sourcing) and measures (e.g., essay writing or sentence verification task) for multiple-texts comprehension, thus results have limited comparability across studies.
iv) The studies included digital or paper texts, but recent studies have suggested that readers' performance may change depending on the medium (see Latini et al., 2019), thus the effect of EFs on multiple-texts comprehension may be moderated by this variable.

When reading multiple texts, in addition to the demands of single-text comprehension (comprehension processes), readers need to represent relevant information about sources and connect it to text content (intertext model achieved through sourcing processes) and construct a representation of the situation described in the texts (integrated situation model, achieved through intertextual integration processes) (Britt et al., 1999; Perfetti et al., 1999).

Following, we review direct evidence on the working memory in creating different types of models (i.e. surface model of single texts, intertext model, and integrated situation model). Overall, the results seem to suggest that working memory is involved in surface comprehension of single texts (Braasch et al., 2014; Mason et al., 2017; Beker et al., 2019). Conversely results about the involvement of working memory at the integrated situation model are mixed (Braasch et al., 2014; Beker et al., 2019; Florit et al., 2020). The studies which assessed intertextual integration share several differences, making it difficult to draw an even tentative interpretation: sample (upper-secondary school students or primary school students); task (prompted open question or recall) and medium (written responses or oral responses). Working memory seems to be partially involved in constructing intertext models by acting on sourcing, especially in evaluation processes (Braasch et al., 2014), but not in justification processes (Braasch et al., 2014; Mason et al., 2018) or sourcing while writing an essay (Florit et al., 2020). Moreover, some evidence suggests that working memory may be involved in creating representations of relevant information about the source, but may not be associated with the epistemic nature of the reader's reflection, that is the justification criteria used to support their trustworthiness judgments.

Problem-solving seems involved in the creation of an integrated mental model. To date, whether one of the main problem-solving phases is more involved than the others is unknown, whereas it could be useful to distinguish the differential role that problem representing, strategy planning, solution implementation and results monitoring play in multiple text comprehension. On a speculative level, this set of results may be interpreted by considering the process of multiple-texts comprehension as a decisional task, mostly tapping on problem-solving skills. Readers need to decide which source is more task-relevant, in which order sources should be read, which intertextual strategies are more appropriate to the specific sources selected. When evaluating sources, different characteristics need to be kept in mind at the same time, which explains the involvement of working memory.

It is unclear whether when reading multiple texts working memory plays a role in maintaining refreshed information in short term memory or rather in updating previous information according to the incoming ones. The distinction between maintaining and updating (Morra et al., 2018) has important implications for cognitive models of working memory and text comprehension, especially on digital media (Harvey and Walker, 2018) and for understanding where children with reading disorders face more difficulties (Borella et al., 2020).

Given the scarcity of the studies reviewed, it is of paramount importance to increase the number of studies that directly investigate the association between working memory and multiple-texts comprehension. To identify promising directions for future research, we reviewed studies that provided indirect evidence on the association between working memory and multiple-texts comprehension. Moreover, we verified whether other EFs were assessed.

### Discussion of Indirect Evidence

A part of the indirect evidence still contributes to our understanding of how working memory may be engaged in multiple-texts comprehension processes. Working memory capacity seems involved with several processes supporting multiple-texts comprehension: epistemic cognition, web searching, text structure identification. These are steps that are well-captured by the RESOLV model (Rouet et al., 2017), according to which multiple-text comprehension depends on the interaction between three levels of representation: the context model, the task model and reading processes and outcomes.

Working memory seems involved in the construction of a context model. This is influenced by the reader's pre-existing context schemata, including epistemic cognition. Working memory was found to be associated with epistemic cognition: higher-performing students in working memory presented a more sophisticated epistemic cognition, that is they justified their perspective referring more to multiple sources and less to personal opinion (Bråten and Ferguson, 2014). This result is in contradiction with what was found by prior studies (Braasch et al., 2014; Mason et al., 2018). However, it must be noticed that Braten and Ferguson assessed epistemic sophistication through an epistemic beliefs questionnaire, whereas in Braasch et al.'s and Mason et al.'s studies epistemic sophistication was measured as “enacted” within a reading task. As the authors themselves claimed, the contribution of working memory may vary across domains and contexts (Bråten and Ferguson, 2014).

Working memory is then associated with two fundamental reading processes: identifying relevant texts (a sourcing process) and identifying the underlying structure of texts (an intertextual integration process). Two studies explored web searching and learning performance (Sharit et al., 2015; Moehring et al., 2016), although not focusing directly on learning from multiple texts. Although working memory capacity has been the most investigated EF in the literature we reviewed, other EFs may be into play, with an even more relevant role. For instance, figural reasoning was found associated with digital literacy (Moehring et al., 2016), and its effect may extend to learning performances when reading digital texts. Reasoning and planning were found to be associated with search accuracy (Sharit et al., 2015), a step that precedes the reading of multiple texts. When surfing the web, readers have to identify task-relevant texts, but even when they are given the texts to study, they can plan the reading order and the time to dedicate to each of them (see Tarchi, 2021). Indeed, readers with high planning and reasoning performances make more transitions between websites and are more accurate when searching for information (Sharit et al., 2015).

Working memory seems to be associated with readers' ability to understand and recall the underlying structure of a series of texts, that is how texts are hierarchically organized (Banas and Sanchez, 2012). Individual differences in working memory capacity uniquely influenced the learning of implicit relationships underlying digital texts within multiple web pages, holding and integrating relevant (but not explicit) information in mind. Of notice, this applies to a context in which readers have to “recognize” a pre-existing structure, as texts were hierarchically organized, rather than create a structure underlying texts without pre-existing connections.

One study provided some evidence supporting the involvement of other EFs than working memory. Brand-Gruwel et al.'s study 2009 seems to suggest that the involvement of the EFs regulation and monitoring when learning from the web may increase with age and expertise. In specific, secondary school students were less able to regulate and monitor their learning activities than older students did, although such a difference did not transfer to learning performances (deep comprehension and source evaluation). In other words, EFs seemed to function at the process-level but not at the product-level. This may happen because people with different profiles may approach the task with different yet equally effective elaborative processes.

## Conclusions

In conclusion, this systematic review emphasizes that the literature on the association between working memory and multiple-texts comprehension is rather fragmented and skewed toward older populations. While it is not possible to draw solid conclusions, we advance here a few indications that can derive from studies and suggestions for future research.

What is the state of art of research on the association between working memory and multiple-text comprehensions? What is the state of art of research investigating the contribution of other EFs? Working memory has received some attention, whereas the other executive functions have been neglected. Some studies have directly assessed problem-solving and fluid intelligence, and indirectly assessed regulation skills, but particularly concerning is the lack of studies on inhibiting and shifting. These two executive functions are considered to be the base of more complex EFs (e.g., Miyake et al., 2000; Diamond, 2013; Friedman and Miyake, 2017; Morra et al., 2018), and have already been theorized to be involved in multiple-texts comprehension (Follmer and Sperling, 2020).

What is the contribution of working memory to the different processes involved in multiple-texts comprehension processes? The evidence from direct and indirect studies suggests an involvement of working memory in intertextual comprehension (Isberner et al., 2013; Braasch et al., 2014; Andresen et al., 2019) in adults. More conflictual is the involvement of working memory with sourcing as it seems involved in document selection (Braasch et al., 2014) but not in sourcing (Mason et al., 2018).

Are there trends in data as a function of age (developmental trends)? It appears that working memory is involved in older students (Isberner et al., 2013; Braasch et al., 2014; Andresen et al., 2019) but not in younger ones (Beker et al., 2019; Florit et al., 2020). These results may depend on the development trajectory of working memory (still emerging in younger students), or on the complexity of tasks (for instance younger kids may have texts available while performing the outcome task, which reduces the demands on working memory). Moreover, Brand-Gruwel et al. (2009) directly tested the developmental hypothesis and found that older participants displayed higher regulation than younger ones.

From the analysis of the gap between the importance played by working memory in learning and evidence currently available in the context of multiple-texts comprehension it is possible to derive three main lines of future research.

What are the trends in data as a function of how constructs are defined? One problem is represented by the vast array of measures used to assess multiple-texts comprehension. Scholars can ask to write argumentative essays, verify whether sentence derive from the texts or not, detect and correct inconsistencies, evaluate the trustworthiness of documents, remember source-content association, search and select documents and many more. This variety provides a complex but not integrated representation of the multiple-texts comprehension construct. Future studies should analyze the effect of working memory on more than one outcome associated with a multiple-texts comprehension task to determine general and specific links across constructs. For instance, Banas and Sanchez's (2012) results suggest an involvement of working memory in the recognition of the structure underlining multiple texts, a crucial process in the construction of an integrated situation model.

How do results on the association between working memory and multiple-text comprehension map on current theoretical frameworks of the latter construct? Direct evidence indicates that working memory contributes to the construction of the integrated situation model theorized in the Documents Model (Britt et al., 1999; Perfetti et al., 1999). Bråten and Ferguson's (2014) results about an association between working memory and epistemic cognition suggests an involvement of the former in determining the readers' default stance by acting on their level of affective engagement (List and Alexander, 2017a). The evidence here reported is anecdotal and it is necessary to conduct more theory-driven research.

The research on the association between working memory and multiple-document comprehension is still at an early stage. Future research should focus more directly on working memory, treating them as an independent variable rather than a mere control variable in the research designs. Moreover, the contribution of inhibition and shifting should also be explored. The hypotheses about the involvement of working memory, inhibition and shifting are speculative and need an evidence base. This type of research is of paramount importance, given the increasing complexity of the contexts in which reading activities take place, with a consequent overburden of readers' cognitive processes.

## Author Contributions

CT and CP contributed to conception of the study, organization of the database, and writing of the draft. CR contributed to the organization of the database and wrote sections of the manuscript. All authors contributed to manuscript revision, read, and approved the submitted version.

## Conflict of Interest

The authors declare that the research was conducted in the absence of any commercial or financial relationships that could be construed as a potential conflict of interest.

## Publisher's Note

All claims expressed in this article are solely those of the authors and do not necessarily represent those of their affiliated organizations, or those of the publisher, the editors and the reviewers. Any product that may be evaluated in this article, or claim that may be made by its manufacturer, is not guaranteed or endorsed by the publisher.

## References

[B1] ^*^ Andresen A. Anmarkrud Ø. Bråten I. (2019). Investigating multiple source use among students with and without dyslexia. Read. Writing 32, 1149–1174. 10.1007/s11145-018-9904-z

[B2] Anmarkrud Ø. Bråten I. Strømsø H. I. (2014). Multiple-documents literacy: strategic processing, source awareness, and argumentation when reading multiple conflicting documents. Learn. Individ. Diff. 30, 64–76. 10.1016/j.lindif.2013.01.007

[B3] ^**^ Banas S. Sanchez C. A. (2012). Working memory capacity and learning underlying conceptual relationships across multiple documents. Appl. Cogn. Psychol. 26, 594–600. 10.1002/acp.2834

[B4] Barzilai S. Eshet-Alkalai Y. (2015). The role of epistemic perspectives in comprehension of multiple author viewpoints. Learn. Instruct. 36, 86–103. 10.1016/j.learninstruc.2014.12.003

[B5] Barzilai S. Tzadok E. Eshet-Alkalai Y. (2015). Sourcing while reading divergent expert accounts: pathways from views of knowing to written argumentation. Instruct. Sci. 43, 737–766. 10.1007/s11251-015-9359-4

[B6] Barzilai S. Zohar A. R. Mor-Hagani S. (2018). Promoting integration of multiple texts: a review of instructional approaches and practices. Educ. Psychol. Rev. 30, 973–999. 10.1007/s10648-018-9436-8

[B7] ^*^ Beker K. van den Broek P. Jolles D. (2019). Children's integration of information across texts: reading processes and knowledge representations. Read. Writing Interdiscipl. J. 663–687. 10.1007/s11145-018-9879-9

[B8] Borella E. Carretti B. Pelegrina S. (2010). The specific role of inhibition in reading comprehension in good and poor comprehenders. J. Learn. Disabil. 43, 541–552. 10.1177/0022219410371676 20606207

[B9] Borella E. Pezzuti L. De Beni R. Cornoldi C. (2020). Intelligence and working memory: evidence from administering the WAIS-IV to Italian adults and elderly. Psychol. Res. 84, 1622–1634. 10.1007/s00426-019-01173-7 30949787

[B10] Bråten I. Britt M. A. Strømsø H. I. Rouet J.-F. (2011). The role of epistemic beliefs in the comprehension of multiple expository texts: toward an integrated model. Educ. Psychol. 46, 48–70. 10.1080/00461520.2011.538647

[B11] ^**^ Bråten I. Ferguson L. E. (2014). Investigating cognitive capacity, personality, and epistemic beliefs in relation to science achievement. Learn. Individ. Diff. 36, 124–130. 10.1016/j.lindif.2014.10.003

[B12] Bråten I. Ferguson L. E. Anmarkrud Ø. Strømsø H. I. (2013). Prediction of learning and comprehension when adolescents read multiple texts: the roles of word-level processing, strategic approach, and reading motivation. Read. Writing 26, 321–348. 10.1007/s11145-012-9371-x

[B13] Bråten I. Stadtler M. Salmerón L. (2018). The role of sourcing in discourse comprehension, in The Routledge Handbook of Discourse Processes, eds Schober M. F. Rapp D. Britt M. A. (NewYork, NY: Routledge), 407.

[B14] ^**^ Bråten I. Strømsø H. I. (2003). A longitudinal think-aloud study of spontaneous strategic processing during the reading of multiple expository texts. Read. Writing 16, 195–218. 10.1023/A:1022895207490

[B15] Bråten I. Strømsø H. I. Britt M. A. (2009). Trust matters: examining the role of source evaluation in students' construction of meaning within and across multiple texts. Read. Res. Q. 44, 6–28. 10.1598/RRQ.44.1.1

[B16] Braasch J. L. G. Bråten I. (2017). The Discrepancy-Induced Source Comprehension (D-ISC) Model: basic assumptions and preliminary evidence. Educ. Psychol. 52, 167–181. 10.1080/00461520.2017.1323219

[B17] ^*^ Braasch J. L. G. Bråten I. Strømsø H. I. Anmarkrud Ø. (2014). Incremental theories of intelligence predict multiple document comprehension. Learn. Individ. Diff. 31, 11–20. 10.1016/J.LINDIF.2013.12.012

[B18] Braasch J. L. G. Bråten I. Strømsø H. I. Anmarkrud Ø. Ferguson L. E. (2013). Promoting secondary school students' evaluation of source features of multiple documents. Contemp. Educ. Psychol. 38, 180–195. 10.1016/j.cedpsych.2013.03.003

[B19] Brand-Gruwel S. Wopereis I. Vermetten Y. (2005). Information problem solving by experts and novices: analysis of a complex cognitive skill. Comput. Hum. Behav. 21, 487–508. 10.1016/j.chb.2004.10.005

[B20] ^**^ Brand-Gruwel S. Wopereis I. Walraven A. (2009). A descriptive model of information problem solving while using internet. Comput. Educ. 53, 1207–1217. 10.1016/j.compedu.2009.06.004

[B21] Bransford J. Stein B. (1993). The Ideal Problem Solver. Centers for Teaching and Technology - Book Library, 46. Available online at: https://digitalcommons.georgiasouthern.edu/ct2-library/46 (accessed March 15, 2021).

[B22] Brante E. W. Strømsø H. I. (2018). Sourcing in text comprehension: a review of interventions targeting sourcing skills. Educ. Psychol. Rev. 30, 773–799. 10.1007/s10648-017-9421-7

[B23] Britt M. A. Perfetti C. A. Sandak R. Rouet J.-F. (1999). Content integration and source separation in learning from multiple texts, in Narrative, Comprehension, Causality, and Coherence: Essays in Honor of Tom Trabasso, eds S. R. Goldman, A. C. Graesser, and P. van den Broek (Mahwah, NJ: Erlbaum), 209–233.

[B24] Bull R. Lee K. (2014). Executive functioning and mathematics achievement. Child Dev. Perspect. 8, 36–41. 10.1111/cdep.12059

[B25] Burin D. I. Barreyro J. P. Saux G. Irrazábal N. C. (2015). Navigation and comprehension of digital expository texts: hypertext structure, previous domain knowledge, and working memory capacity. Electron. J. Res. Educ. Psychol. 13, 529–550. 10.14204/ejrep.37.14136

[B26] Butterfuss R. Kendeou P. (2018). The role of executive functions in reading comprehension. Educ. Psychol. Rev. 30, 801–826. 10.1007/s10648-017-9422-620200841

[B27] Carretti B. Cornoldi C. De Beni R. Romanò M. (2005). Updating in working memory: a comparison of good and poor comprehenders. J. Exp. Child Psychol. 91, 45–66. 10.1016/j.jecp.2005.01.005 15814095

[B28] Cartwright K. B. (2015). Executive function and reading comprehension: the critical role of cognitive flexibility, in Comprehension Instruction: Research-Based Best, eds S. R. Parris and K. Headley (New York, NY: Guilford), 56–71.

[B29] Christopher M. E. Miyake A. Keenan J. M. Pennington B. Defries J. C. Wadsworth S. J. . (2012). Predicting word reading and comprehension with executive function and speed measures across development: a latent variable analysis. J. Exp. Psychol. Gen. 141, 470–488. 10.1037/a0027375 22352396 PMC3360115

[B30] Craig F. Margari F. Legrottaglie A. R. Palumbi R. de Giambattista C. Margari L. (2016). A review of executive function deficits in autism spectrum disorder and attention-deficit/hyperactivity disorder. Neuropsychiatr. Dis. Treat. 12, 1191–1202. 10.2147/NDT.S104620 27274255 PMC4869784

[B31] Craik F. (1986). A functional account of age differences in memory, in Human Memory and Cognitive Capabilities, Mechanisms, and Performances, eds F. Kilx and H. Hagendorf (North-Holland, NH: Elsevier Science), 409–422.

[B32] D'Amico A. Passolunghi M. C. (2009). Naming speed and effortful and automatic inhibition in children with arithmetic learning disabilities. Learn. Individ. Diff. 19, 170–180. 10.1016/j.lindif.2009.01.001

[B33] Daneman M. Carpenter P. A. (1980). Individual differences in working memory and reading. J. Verb. Learn. Verb. Behav. 19, 450–466. 10.1016/s0022-5371(80)90312-6

[B34] De La Paz S. Felton M. K. (2010). Reading and writing from multiple source documents in history: effects of strategy instruction with low to average high school writers. Contemp. Educ. Psychol. 35, 174–192. 10.1016/j.cedpsych.2010.03.001

[B35] Diamond A. (2013). Executive functions. Annu. Rev. Psychol. 64, 135–168. 10.1146/annurev-psych-113011-143750 23020641 PMC4084861

[B36] Eisenberg M. B. Berkowitz R. E. (1990). Information Problem Solving: The Big Six Skills Approach to Library and Information Skills Instruction. Norwood, NJ: Ablex Publishing Corporation.

[B37] Ekstrom R. B. French J. W. Harman H. H. Dermen D. (1976). Manual for Kit of Factor-Referenced Cognitive Tests. Princeton, NJ: Educational Testing Service.

[B38] ^*^ Florit E. Cain K. Mason L. (2020). Going beyond children's single-text comprehension: the role of fundamental and higher-level skills in 4th graders' multiple-document comprehension. Brit. J. Educ. Psychol. 90, 449–472. 10.1111/bjep.12288 31070262

[B39] Follmer D. J. (2018). Executive function and reading comprehension: a meta-analytic review. Educ. Psychol. 53, 42–60. 10.1080/00461520.2017.1309295

[B40] Follmer D. J. Sperling R. A. (2020). The roles of executive functions in learning from multiple representations and perspectives, in Handbook of Learning from Multiple Representations and Perspectives, eds P. Van Meter, A. List, D. Lombardi, and P. Kendeou (New York, NY: Routledge), 297–313.

[B41] Frederick S. (2005). Cognitive reflection and decision making. J. Econ. Perspect. 19, 25–42. 10.1257/089533005775196732

[B42] Friedman N. P. Miyake A. (2017). Unity and diversity of executive functions: individual differences as a window on cognitive structure. Cortex 86, 186–204. 10.1016/j.cortex.2016.04.023 27251123 PMC5104682

[B43] Garcia R. B. Tomaino A. Cornoldi C. (2019). Cross-modal working memory binding and learning of visual-phonological associations in children with reading difficulties. Child Neuropsychol. 25, 1063–1083. 10.1080/09297049.2019.1572729 30706757

[B44] Georgiou G. K. Das J. P. (2016). What component of executive functions contributes to normal and impaired reading comprehension in young adults? Res. Dev. Disabil. 49–50, 118–128. 10.1016/j.ridd.2015.12.001 26704777

[B45] Gil L. Bråten I. Vidal-Abarca E. Strømsø H. I. (2010). Summary versus argument tasks when working with multiple documents: which is better for whom? Contemp. Educ. Psychol. 35, 157–173. 10.1016/j.cedpsych.2009.11.002

[B46] Goldman S. R. Braasch J. L. G. Wiley J. Graesser A. C. Brodowinska K. (2012). Comprehending and learning from internet sources: processing patterns of better and poorer learners. Read. Res. Q. 47, 356–381. 10.1002/RRQ.027

[B47] Guajardo N. R. Cartwright K. B. (2016). The contribution of theory of mind, counterfactual reasoning, and executive function to pre-readers' language comprehension and later reading awareness and comprehension in elementary school. J. Exp. Child Psychol. 144, 27–45. 10.1016/j.jecp.2015.11.004 26689129

[B48] Harvey H. Walker R. (2018). Reading comprehension and its relationship with working memory capacity when reading horizontally scrolling text. Q. J. Exp. Psychol. 71, 1887–1897. 10.1080/17470218.2017.1363258 28766371

[B49] Hasher L. Zacks R. T. Rahhal T. A. (1999). Timing, instructions, and inhibitory control: some missing factors in the age and memory debate. Gerontology 45, 355–357. 10.1159/000022121 10559658

[B50] Hilbert T. S. Renkl A. (2008). Concept mapping as a follow-up strategy to learning from texts: what characterizes good and poor mappers? Instruct. Sci. 36, 53–73. 10.1007/s11251-007-9022-9

[B51] ^**^ Isberner M.-B. Richter T. Maier J. Knuth-Herzig K. Horz H. Schnotz W. (2013). Comprehending conflicting science-related texts: graphs as plausibility cues. Instruct. Sci. 49, 849–872. 10.1007/s11251-012-9261-2

[B52] Johann V. Könen T. Karbach J. (2020). The unique contribution of working memory, inhibition, cognitive flexibility, and intelligence to reading comprehension and reading speed. Child Neuropsychol. 26, 324–344. 10.1080/09297049.2019.1649381 31380706

[B53] Kassai R. Futo J. Demetrovics Z. Takacs Z. K. (2019). A meta-analysis of the experimental evidence on the near- and far-transfer effects among children's executive function skills. Psychol. Bull. 145, 165–188. 10.1037/bul0000180 30652908

[B54] Kendeou P. Smith E. R. O'Brien E. J. (2013). Updating during reading comprehension: why causality matters. J. Exp. Psychol. Learn. Mem. Cogn. 39, 854–865. 10.1037/a0029468 22845069

[B55] Kobayashi K. (2015). Learning from conflicting texts: the role of intertextual conflict resolution in between-text integration. Read. Psychol. 36, 519–544. 10.1080/02702711.2014.926304

[B56] ^*^ Latini N. Bråten I. Anmarkrud Ø. Salmerón L. (2019). Investigating effects of reading medium and reading purpose on behavioral engagement and textual integration in a multiple text context. Contemp. Educ. Psychol. 59, 101797. 10.1016/J.CEDPSYCH.2019.101797

[B57] Li M. Murphy P. K. Wang J. Mason L. H. Firetto C. M. Wei L. . (2016). Promoting reading comprehension and critical-analytic thinking: a comparison of three approaches with fourth and fifth graders. Contemp. Educ. Psychol. 46, 101–115. 10.1016/j.cedpsych.2016.05.002

[B58] List A. Alexander P. A. (2017a). Cognitive affective engagement model of multiple source use. Educ. Psychol. 52, 182–199. 10.1080/00461520.2017.1329014

[B59] List A. Alexander P. A. (2017b). Analyzing and integrating models of multiple text comprehension. Educ. Psychol. 52, 143–147. 10.1080/00461520.2017.1328309

[B60] List A. Alexander P. A. (2019). Toward an integrated framework of multiple text use. Educ. Psychol. 1, 20–39. 10.1080/00461520.2018.1505514

[B61] Mammarella I. C. Lucangeli D. Cornoldi C. (2010). Spatial working memory and arithmetic deficits in children with nonverbal learning difficulties. J. Learn. Disabil. 43, 455–468. 10.1177/0022219409355482 20375290

[B62] Mammarella I. C. Meneghetti C. Pazzaglia F. Cornoldi C. (2015). Memory and comprehension deficits in spatial descriptions of children with non-verbal and reading disabilities. Front. Psychol. 5:1534. 10.3389/fpsyg.2014.01534 25610417 PMC4285864

[B63] ^*^ Mason L. Scrimin S. Tornatora M. C. Suitner C. Moè A. (2018). Internet source evaluation: the role of implicit associations and psychophysiological self-regulation. Comput. Educ. 119, 59–75. 10.1016/j.compedu.2017.12.009

[B64] ^*^ Mason L. Scrimin S. Tornatora M. C. Zaccoletti S. (2017). Emotional reactivity and comprehension of multiple online texts. Learn. Individ. Diff. 58, 10–21. 10.1016/J.LINDIF.2017.07.002

[B65] Mateos M. Cuevas I. Martín E. Martín A. Echeita G. Luna M. (2011). Reading to write an argumentation: the role of epistemological, reading and writing beliefs. J. Res. Read. 34, 281–297. 10.1111/j.1467-9817.2010.01437.x

[B66] Mateos M. Martín E. Cuevas I. Villalón R. Martínez I. González-Lamas J. (2018). Improving written argumentative synthesis by teaching the integration of conflicting information from multiple sources. Cogn. Instruct. 36, 119–138. 10.1080/07370008.2018.1425300

[B67] Miyake A. Friedman N. P. (2012). The nature and organization of individual differences in executive functions. Curr. Direct. Psychol. Sci. 21, 8–14. 10.1177/0963721411429458 22773897 PMC3388901

[B68] Miyake A. Friedman N. P. Emerson M. J. Witzki A. H. Howerter A. Wager T. D. (2000). The unity and diversity of executive functions and their contributions to complex “frontal lobe” tasks: a latent variable analysis. Cogn. Psychol. 41, 49–100. 10.1006/COGP.1999.0734 10945922

[B69] ^**^ Moehring A. Schroeders U. Leichtmann B. Wilhelm O. (2016). Ecological momentary assessment of digital literacy: influence of fluid and crystallized intelligence, domain-specific knowledge, and computer usage. Intelligence 59, 170–180. 10.1016/j.intell.2016.10.003

[B70] Moher D. Liberati A. Tetzlaff J. Altman D. G. (2009). Preferred reporting items for systematic reviews and meta-analyses: the PRISMA statement. PLoS Med. 6:e1000097. 10.1371/journal.pmed.1000097 19621072 PMC2707599

[B71] Morra S. Panesi S. Traverso L. Usai M. C. (2018). Which tasks measure what? Reflections on executive function development and a commentary on Podjarny, Kamawar, and Andrews (2017). J. Exp. Child Psychol. 167, 246–258. 10.1016/j.jecp.2017.11.004 29197781

[B72] Muijselaar M. M. L. de Jong P. F. (2015). The effects of updating ability and knowledge of reading strategies on reading comprehension. Learn. Individ. Diff. 43, 111–117. 10.1016/j.lindif.2015.08.011

[B73] Oberauer K. Süß H. M. Schulze R. Wilhelm O. Wittmann W. W. (2000). Working memory capacity - Facets of a cognitive ability construct. Pers. Individ. Diff. 29, 1017–1045. 10.1016/S0191-8869(99)00251-2

[B74] Palladino P. Cornoldi C. De Beni R. Pazzaglia F. (2001). Working memory and updating processes in reading comprehension. Mem. Cogn. 29, 344–354. 10.3758/BF03194929 11352218

[B75] Pazzaglia F. Palladino P. De Beni R. (2000). Presentazione di uno strumento per la valutazione della memoria di lavoro verbale e sua relazione con i disturbi della comprensione (en. tr. Presentation of an instrument to assess verbal working memory and its relation with reading comprehension disorde). Psicologia Clinica dello Sviluppo 4, 465–486. 10.1449/608

[B76] Pereira A. Lopes S. Magalhães P. Sampaio A. Chaleta E. Rosário P. (2018). How executive functions are evaluated in children and adolescents with cerebral palsy? A systematic review. Front. Psychol. 9:21. 10.3389/fpsyg.2018.00021 29467685 PMC5808176

[B77] Perfetti C. A. Rouet J.-F. Britt M. A. (1999). Toward a theory of documents representation, in The Construction of Mental Representation During Reading, eds H. Van Oostendorp and S. R. Goldman (Mahwah, NJ: Erlbaum), 99–122.

[B78] Pressley M. Afflerbach P. (1995). Verbal Protocols of Reading: The Nature of Constructively Responsive Reading. Hillsdale, NJ: Erlbaum.

[B79] Primor L. Katzir T. (2018). Measuring multiple text integration: a review. Front. Psychol. 9:2294. 10.3389/fpsyg.2018.02294 30555372 PMC6282655

[B80] Reitan R. M. (1958). Validity of the trail making test as an indication of organic brain damage. Percept. Motor Skills 8, 271–276.

[B81] Richter T. Maier J. (2017). Comprehension of multiple documents with conflicting information: A two-step model of validation. Educ. Psychol. 52, 148–166. 10.1080/00461520.2017.1322968

[B82] Rouet J.-F. Britt M. A. (2011). Relevance processes in multiple document comprehension, in Text Relevance and Learning from Text, eds M. T. McCrudden, J. P. Magliano, and G. Schraw (Charlotte, NC: Information Age Publishing), 19–52.

[B83] Rouet J.-F. Britt M. A. Durik A. M. (2017). RESOLV: readers' representation of reading contexts and tasks. Educ. Psychol. 52, 200–215. 10.1080/00461520.2017.1329015

[B84] ^**^ Sharit J. Taha J. Berkowsky R. W. Profita H. Czaja S. J. (2015). Online information search performance and search strategies in a health problem-solving scenario. J. Cogn. Eng. Decis. Mak. 9, 211–228. 10.1177/1555343415583747 29056885 PMC5650100

[B85] Swanson H. L. Trahan M. F. (1992). Learning disabled readers' comprehension of computer mediated text: the influence of working memory, metacognition and attribution. Learn. Disabil. Res. Pract. 7, 74–86.

[B86] Swanson H. L. Xinhua Z. Jerman O. (2009). Working memory, short-term memory, and reading disabilities: a selective meta-analysis of the literature. J. Learn. Disabil. 42, 260–287. 10.1177/0022219409331958 19255286

[B87] Tarchi C. (2021). Prompting readers to plan might negatively affect their comprehension of multiple documents. J. College Read. Learn. 51, 131–152. 10.1080/10790195.2020.1823910

[B88] Unsworth N. Heitz R. P. Schrock J. C. Engle R. W. (2005). An automated version of the operation span task. Behav. Res. Methods 37, 498–505. 10.3758/BF03192720 16405146

[B89] van Dijk T. A. Kintsch W. (1983). Strategies of Discourse Comprehension. New York, NY: Academic Press.

[B90] van Laar E. van Deursen A. J. A. M. van Dijk J. A. G. M. de Haan J. (2017). The relation between 21st-century skills and digital skills: a systematic literature review. Comput. Hum. Behav. 72, 577–588. 10.1016/j.chb.2017.03.010

[B91] van Someren M. W. Barnard Y. F. Sandberg J. A. C. (1994). The Think Aloud Method. A Practical Guide to Modelling Cognitive Processes. London: Academic Press.

[B92] Viterbori P. Usai M. C. Traverso L. De Franchis V. (2015). How preschool executive functioning predicts several aspects of math achievement in Grades 1 and 3: a longitudinal study. J. Exp. Child Psychol. 140, 38–55. 10.1016/j.jecp.2015.06.014 26218333

[B93] Wechsler D. (1981). The Digit Symbol Substitution Test: Wechsler Adult Intelligence Scale–Revised. New York, NY: Psychological Corporation.

[B94] Weinstein C. E. Husman J. Dierking D. R. (2000). Self-regulation interventions with a focus on learning strategies, in Self-Regulation: Theory, Research, and Applications, eds M. Boekaerts, P. Pintrich, and M. Seidner (Orlando, FL: Academic Press), 727–747.

[B95] Wiley J. Goldman S. R. Graesser A. C. Sanchez C. A. Ash I. K. Hemmerich J. A. (2009). Source evaluation, comprehension, and learning in internet science inquiry tasks. Am. Educ. Res. J. 46, 1060–1106. 10.3102/0002831209333183

[B96] Wilhelm O. Schroeders U. Schipolowski S. (2014). Berliner Test zur Erfassung fluider und kristalliner Intelligenz für die 8. bis 10. Jahrgangsstufe (BEFKI 8-10) (en. tr. “Berlin test for the assessment of fluid and crystalized intelligence in 8-10 year-old children”). Goettingen: Hogrefe.

[B97] Zelazo P. D. Carlson S. M. (2020). The neurodevelopment of executive function skills: implications for academic achievement gaps. Psychol. Neurosci. 13, 273–298. 10.1037/pne0000208

[B98] Zelazo P. D. Carter A. Reznick J. S. Frye D. (1997). Early development of executive function: a problem-solving framework. Rev. Gen. Psychol. 1, 198–226. 10.1037/1089-2680.1.2.198

